# Immunomagnetic separation and Listeria monocytogenes detection with surface-enhanced Raman scattering

**DOI:** 10.3906/sag-2002-234

**Published:** 2020-06-23

**Authors:** Hande YEĞENOĞLU AKÇINAR, Belma ASLIM*, Hilal TORUL, Burcu GÜVEN, Adem ZENGİN, Zekiye SULUDERE, İsmail Hakkı BOYACI, Uğur TAMER

**Affiliations:** 1 Department of Biology, Faculty of Science, Gazi University, Ankara Turkey; 2 Department of Analytical Chemistry, Faculty of Pharmacy, Gazi University, Ankara Turkey; 3 Department of Food Engineering, Faculty of Engineering, Hacettepe University, Ankara Turkey; 4 Department of Chemical Engineering, Faculty of Engineering, Yüzüncü Yıl University, Van Turkey

**Keywords:** Immunomagnetic separation (IMS), surface-enhanced Raman scattering (SERS), *Listeria monocytogenes*(*L. monocytogenes*)

## Abstract

**Background/aim:**

We aimed to develop a rapid method to enumerate *Listeria monocytogenes* (*L. monocytogenes*) utilizing magnetic nanoparticle based preconcentration and surface-enhanced Raman spectroscopy measurements.

**Materials and methods:**

Biological activities of magnetic Au-nanoparticles have been observed to have the high biocompatibility, and a sample immunosensor model has been designed to use avidin attached Au-nanoparticles for *L. monocytogenes *detection. *Staphylococcus aureus* (*S. aureus*) and *Salmonella typhimurium* (*S. typhimurium*) bacteria cultures were chosen for control studies. Antimicrobial activity studies have been done to identify bio-compatibility and bio-characterization of the Au-nanoparticles in our previous study and capturing efficiencies to bacterial surfaces have been also investigated.

**Results:**

We constructed the calibration graphs in various population density of *L. monocytogenes *as 2.2 × 101 to 2.2 × 106 cfu/mL and the capture efficiency was found to be 75%. After the optimization procedures, population density of *L. monocytogenes *and Raman signal intensity showed a good linear correlation (R2 = 0.991) between 102 to 106 cfu/mL *L. monocytogenes*. The presented sandwich assay provides low detection limits and limit of quantification as 12 cfu/mL and 37 cfu/mL, respectively. We also compared the experimental results with reference plate-counting methods and the practical utility of the proposed assay is demonstrated using milk samples.

**Conclusion:**

It is focused on the enumeration of *L. monocytogenes *in milk samples and the comparision of results of milk analysis obtained by the proposed SERS method and by plate counting method stay in food agreement. In the present study, all parameters were optimized to select SERS-based immunoassay method for *L. monocytogenes* bacteria to ensure LOD, selectivity, precision and repeatablity.

## 1. Introduction

*Listeria monocytogenes* (*L. monocytogenes*) is a crucial foodborne pathogenentailing disease. *L. monocytogenes *can grow and develop even at refrigerator temperatures and is a major problem, especially in ready-to-eat foods. Listeriosis illness is caused by contaminated foods with *L. monocytogenes *[1]. Raw milk is known as an important source of *L. monocytogenes*. In 1986, Hayes et al. isolated this bacterium from 12 samples from 100 raw milk samples in USA [2]. 

Rapid pathogenic bacterial diagnosis has been applied to conduct measurements in biological and food matrix [3]. Up to date, different method has been applied by several research group for enumeration of pathogenic bacteriaespecially *L. monocytogenes* using polymerase chain reaction immunoassay [4,5], electrochemical sensors [6–8], bioluminescence [9,10], DNA-based sensors [u2935], ELISA [u2936], surface plasmon resonance [u2937], fluorescence [u2938], surface-enhanced Raman scattering (SERS) [19–21]. It was indicated that the reported methods were optimized to select proper system usage to obtain selectivity and precision, there were some problems such as poor sensitivity and long experimental procedures. Also, the enumeration of pathogenin food matrix is problematic [22]. Therefore, new analytical methods are required for the detecting of pathogens and other biomolecules in food matrix. Recently, immunomagnetic separation (IMS) overcomes the matrix effect and is used for the enumeration of bacteria. IMS can eliminate the potential interferences and it has been applied to conduct measurements in food matrix, thereby bacteria can be captured easily [23,24]. 

In recent years, SERS is commonly used due to its high sensitivity (single molecules can be detected), ability to analyse multiple analytes in one sample, small sample volume, selective to target molecule signal [25–27]. More target molecule can be detected with using the combination of SERS and IMS techniques. Furthermore, the usage of a SERS tag as 5,5’-dithiobis(2-nitrobenzoic acid [28–30], rhodamine dye [31], Texas red [32] enhances the SERS signal and can reach low detection limits compared to label-free detection methods [33,34].

The biocompatibility of nanomaterials in biological systems was characterized and thus, it was aimed to increase the usage possibilities of these nanoparticles. In this study, biological characterization studies such as antimicrobial, antioxidant activities, cytotoxic and anticarcinogenic effects, genotoxicity tests and capturing efficiencies of nanoparticles which would be used as immunoassay design were conducted. In the first part, some parameters (antioxidant activities, cytotoxic, anticarcinogenic effects and genotoxicity tests) of this study were given in our previous study [35]. As a continuation study, antimicrobial characterization and capturing efficiency studies of nanoparticles were performed and the bioassay design of *L. monocytogenes *was developed. In order to determine the antimicrobial effects of nanoparticles, the studies were performed with *L. monocytogenes*, *S. aureus*, *S. typhimurium *bacteria and a liveness rate of approximately 96% was reached on each bacterium and thus, the antimicrobial effects of the magnetic Au-nanospheres were shown to be quite low. The competitive and noncompetitive capturing amount of nanoparticles on bacteria were also studied. The competitive capturing efficiency of magnetic-Au-nanoparticles was found as 75% in immunoassay model. In the noncompetitive studies, the attachment ratio of *L. monocytogenes* was found as higher than the attachment of *S. aureus* and *S. typhimurium*. Then, SERS-based immunoassay method was developed using Au-nanorods (for SERS labeling) and magnetic Au-nanospheres (for IMS). A calibration curve was constracted for the enumaration of *L. monocytogenes *in a model system. The present paper is focused on the enumeration of *L. monocytogenes *in milk samples and the comparision of results of milk analysis obtained by the proposed SERS method and by plate counting method stay in food agreement. In the present study, all parameters were optimized to select SERS-based immunoassay method for *L. monocytogenes* bacteria to ensure LOD, selectivity, precision and repeatablity. 

## 2. Experimental

### 2.1. Materials

Disodium hydrogen phosphate (Na2HPO4), silver nitrate (AgNO3), sodium borohydride (NaBH4), solution (30%), absolute ethanol, perchloric acid, ethanolamine, iron (II) sulfate heptahydrate were purchased from Merck KGaA (Darmstadt, Germany). N-Hydroxysulphosuccinimide sodium salt (NHS) was purchased from Pierce Biotechnology (Bonn, Germany). NaCl, Na2HPO4, and KH2PO4 were purchased from J.T. Baker (Deventer, Netherlands). Hydrogen tetrachloroaurate (HAuCl4), was purchased from Sigma-Aldrich Chemie GmbH (Steinheim, Germany). Other chemicals are analytical grade.

### 2.2. Buffers

Physiological saline (PS) (0.875g/100mL) was prepared by NaCl and distilled water. Na2HPO4, KH2PO4, and NaCl were used for the preparation of PBS buffers (0.1 M, pH 7.4) and adjusted the pH with HCl or NaOH. To adjust the pH of MES buffer (0.05 M, pH 6.5), 0.1 N NaOH was used. The same buffer was also used for the preparation of avidin (0.5 mg/mL). Gluteraldehyde (2.5%) and Osmium tetraoxide (0.1%) were prepared with PS solution (0.875g/100mL). Milli-Q quality water (18 MΩ cm) was used throughout the study.

### 2.3. Microorganisms

*Staphylococcus aureus* (*S. aureus*), *Listeria monocytogenes* (*L. monocytogenes*), *Salmonella typhimurium* (*S. typhimurium*) bacteria cultures were received from Biotechnology Laboratory at Gazi University, Ankara, Turkey. For *L. monocytogenes*, *S. aureus*, *S. typhimurium* detection nutrient broth was purchased from Merck KGaA (Darmstadt, Germany). *L. monocytogenes* colonies were selected easily by using CHROMagarTM Listeria culture medium (CHROMagar Microbiology, Paris, France Listeria). We diluted cultures serially (10-fold steps) with PS buffer and plated with 100 μL diluted solution of the culture. We counted colonies after incubation at 37 °C for 24 h.

### 2.4. Instrumentation

Absorbance measurements of nanoparticles were obtained with an UV-Visible spectrophotometer (Agilent Technologies, Inc., Santa Clara, CA, USA). The Tecnai G2 F30 instrument (FEI Company, Hillsboro, OR, USA) was used to capture TEM images at operated 120 kV. For TEM measurements, 10 μL of nanoparticle solution was dropped and waited for 10 min. FEI Nova NanoSEM 430 microscope (FEI, Eindhoven, Netherlands) was used to get SEM images. Bacteria concentrations were adjusted using a Densitometer (Grant Instruments Ltd., Cambridge, UK). Raman measurements were performed using a Raman Microscopy (Deltanu Inc., Laramie, WY, USA). In the present study, laser source is 785 nm and 20x objective, 30 mm laser spot size, 0.15 W laser power, and 20 s acquisition time.

### 2.5. Fabrication of Au-coated magnetic spherical nanoparticles

In our previous work, we synthesized a core-shell Au@Fe3O4 nanoparticles. Here, with a brief modification, FeCl3 (1.28 M) and FeSO4.7H2O (0.64 M) were prepared and a solution of 1 M NaOH was added dropwise into the mixture with stirring for 40 min. After addition of 1M NaOH, black participate was obtained. This participate was removed from the reaction chamber via simple magnet and washed 3 times. To coat gold layer onto the iron nanoparticles, we performed the same procedure as reported our previous report (37). 

### 2.6. Fabrication of Au-nanorods

For the SERS tag, we synthesized rod shaped Au nanoparticles based on our previous report. Briefly, we prepared a seed solution mixing CTAB (7.5 mL, 0.1 M) and HAuCl4 (250 µL, 0.01 M) solution. Then, we added NaBH4 (ice-cold, 600µL, 0.01 M) to the resulting solution. After waiting for 5 min, CTAB (4.75 mL, 0.1 M), HAuCl4 (1.0 mL, 0.01 M) and AgNO3 (60µL, 0.004 M) were mixed and the orange colour solution was observed. After adding of ascorbic acid (250µL, 0.01 M), the colour turned colourless. Finally, 5 µL seed solution was added to the resulting solution and waited for 3 h.

### 2.7. Immunomagnetic separation (IMS)with modified magnetic nanoparticles

We modified the gold coated magnetic nanoparticles using 0.15 M 11-MUA to form a SAM in ethanol overnight. Then, we collected the nanoparticles using a permanent magnet. EDC/NHS (1 mL) was added to the nanoparticle solution and waited for 40 min. After washing steps (2 times), 50 mM MES buffer solution was added. To modify with avidin, the resulting nanoparticles were incubated with avidin solution for 40 min. To eliminate the nonspecific interactions, we used 1% (v/v) ethanolamine for 1 hour. Then, the biotin-labeled *L. monocytogenes* antibody was added to the avidin modified nanoparticle solution. Then, washing procedure was carried out using PBS to remove unconjugated biotinylated antibodies. All washing procedures have been conducted in an ultrasound bath for 10 s.

### 2.8. Determination of nanoparticles’ antimicrobial activities 

Two different methods were used to determine the antimicrobial activity. In the first (direct) method, antimicrobial activities of magnetic Au-nanoparticles on *L. monocytogenes*, *S. aureus* and *S. typhimurium* strains were tested directly. Each bacteria culture was activated twice in nutrient broth before use. All activated bacteria (*L. monocytogenes*, 6.8 × 107 cfu/mL; *S. aureus*, 10.4 × 1010 cfu/mL; *S. typhimurium*, 7.2 × 107 cfu/mL) concentrations were adjusted to 0.5 McFarland scale using McFarland device and next prepared sterile, nutrient broth was injected with 100 µL bacteria, and this was used as control. In another nutrient broth 100 µL bacterial solution was added with nanoparticle solution having 1 mg/mL in 100 µL. All these mixtures were treated at 37°C for 24 h. The liveness was indicated with inoculation the bacteria on the nutrient agar and the results were also compared with control cultures.

In the second (indirect) method *L. monocytogenes*, *S. aureus* and *S. typhimurium* strains were activated twice and later their concentrations were adjusted separately using McFarland device (Grant-bio, DEN1) to 0.5 McFarland scale using McFarland device (*L. monocytogenes*, 6.8 × 107 cfu/mL; *S. aureus*, 10.4 × 1010 cfu/mL; *S. typhimurium*, 7.2 × 107cfu/mL). 1% (v/v) of bacteria cultures was inoculated into the nutrient broths which contain 1.5% agar. Agar was used as a solidifying agent. After solidifying the medium, the holes were punched with a cork borer in plates of nutrient agar. The holes were then filled with a solution of 25 µL of nanoparticle solution having of 1 mg/mL concentration. The incubation was applied for 24 h at 37 °C and the diameter of clear zones surrounding the wells were determined and indicated the antibacterial activity [36]. All antimicrobial studies were performed with 5 parallel and 2 replicates.

### 2.9. Determination of nanoparticles’ capturing efficiencies

The capturing efficiency studies were performed with avidin coated nanoparticles. Each of the bacteria was activated twice and used in these experiments.The experiments were conducted in mixed culture media including the control medium in order to both determine the adhesion of various pathogenic microorganisms on the avidin coated nanoparticle surfaces and the success of the immunoassay which is specific for *L. monocytogenes* antibody bound nano surfaces. For this purpose, studies were conducted to determine the nanoparticles’ capturing efficiencies of each bacterium in competitive and noncompetitive systems. 

#### 2.9.1. Determination of noncompetitive capturing efficiencies 

In all the capturing efficiency studies, the concentration of nanoparticles was adjusted to 0.5 mg/mL in sterile PS solution. The bacteria were activated twice, and the active cultures were obtained after centrifuge at 10,000 rpm for 15 min and washed and resuspended in PS solution. All activated bacteria (*L. monocytogenes*, 4.6 × 107 cfu/mL; *S. aureus*, 9.6 × 1010 cfu/mL; *S. typhimurium*, 4.4 × 107 cfu/mL) concentrations were adjusted to 0.5 McFarland scale using McFarland device. Then, 0.5 mg/mL nanoparticles were transferred to the bacteria medium and waited for 30 min for incubation. After incubation period, a magnet was used to collect the modified nanoparticles and washing procedure was applied 2 times with PS solution. In the present study, we performed a plate counting methodin the supernatant to determine the capture efficiency by plating the unbound bacteria.

#### 2.9.2. Determination of competitive capturing efficiencies 

In order to determination of the competitive capturing efficiencies, 2 experiments were performed. In the first study, the capturing amounts of avidin modified nanoparticles (unmodified with *L. monocytogenes* antibody) of mixed cultures where *L. monocytogenes* and *S. typhimurium* were present in the A medium and *L. monocytogenes* and *S. aureus* were in the B medium were investigated. In another study, it was designed to test the success of immunoassay detection of *L. monocytogenes* and the capturing amounts of *L. monocytogenes* antibody modified nanoparticles of mixed cultures where *L. monocytogenes* and *S. typhimurium* were present in the A medium and *L. monocytogenes* and *S. aureus* were in the B medium were also investigated.

The concentration of avidin modified nanoparticles was mixed medium containing *L. monocytogenes *and *S. typhimurium* and *L. monocytogenes* and *S. aureus* in sterile PS solution. The bacteria were activated twice, and centrifugation procedure was applied at 10,000 rpm for 15 min. Then, resulting cultures were washed and resuspended in PS solution. All activated bacteria concentrations were adjusted to 1 McFarland scale. 1 mL of each culture was added in a sterile tube to form a new mixed culture. Then, 2 mL of this mixed bacterial culture and 2 mL of 0.5 mg/mL nanoparticle concentration were taken into a new sterile tube and placed in a dark medium for 30 min. Afterwards, a permanent magnet was used to collect nanoparticles and nanoparticles were washed twice with PS solution. Thus, the liveness values ​​of the bacteria that the only attached to the nanoparticle surfaces were calculated in cfu/mL using the CHROMagar Listeria.

In the developed immunosensor, we treated nanoparticles with bacterial cells and the capturing amount of *L. monocytogenes *on the magnetic Au-nanoparticles was shown using SEM and TEM images. For this purpose, 2% of *L. monocytogenes* cultures were inoculated into nutrient medium and incubation was performed at 37 °C for 24 h. After being activated twice, and centrifugation was performed at 5,000 rpm for 10 min and washed and transformed to the PS. All activated bacteria concentrations were adjusted to 0.5 McFarland scale. 100 µL of each culture was added in a 900 sterile µL PS. Here, 0.5 mg/mL nanoparticle solution was transferred to the diluted bacteria medium and incubated for 30 min at room temperature. Then, a magnet was used to obtain bacteria bounded nanoparticles and washing procedures were applied twice with PS solution. TEM images were captured by dropping nanoparticle-bacteria complex (10 µL) using formvar–carbon coated cupper grids and waited for 10 min. 

After adjusting to 0.5 McFarland scale, SEM images were captured to obtain control (*L. monocytogenes* without nanoparticle) and immunoassay model with *L. monocytogenes*. 

Briefly, we applied IMS and collected all bacterial cells interacted with nanoparticles. Then, glutaraldehyde (2.5%) was added to the cell suspensions for fixation procedure at 4 ℃ and waited overnight. After fixation procedure, the cells were pelleted and washed in PBS buffer. Then, we immersed the pellet in osmium tetroxide (1%) in buffer for postfixation procedure. After washing steps with PBS and water for 10 min each, different ethanol concentrations (initial value from 30 mL/100 mL to 100 mL/100 mL) were used for dehydration during 15 min. After applying three 10 min washing procedure with ethanol (100 g/100 g), dehydration process was achieved. To capture SEM images, air-dried SEM stubs were used to form a layer using gold sputter. Here, 10 µL sample was transferred on SEM stubs. In the present study, SEM was used with an acceleration voltage of 10 kV.

### 2.10. Preparation of SEM tag

We performed SERS measurements based on labelled sandwich immunoassay. For this purpose, we synthesized goldnanorods modified with DTNB. Here, 50 mM DTNB was dissolved in ethanol and interacted with gold nanorod for 18 h at room temperature. After washing step with MES buffer (50 mM) for 3 times, centrifugation was applied at 7000 rpm for 5 min. Subsequently, the labelled nanoparticles were taken into 1 mL of MES buffer. 

### 2.11. Detection of L. monocytogenes

A sandwich complex was obtained in a solution phase by interacted with magnetic gold nanospheres with *L. monocytogenes *and DTNB modified gold nanorods. The resulting sandwich complex was interacted for half an hour. Then, a permanent magnet was used to collect the complex. To gain SERS signals from the resulting sandwich complex, we dropped it onto chromatography paper and SERS measurements were conducted 3 times. The SERS spectra corresponding to *L. monocytogenes *were collected. The calibration curve was constructed by obtaining the average SERS reading of *L. monocytogenes *(101–107 cfu/mL). The enumeration was completed by counting the number of colonies plating on CHROMagar Listeria agar subsequent incubation at 37 °C for 24 h. The peak signal intensity at 1336 cm−1 was selected for the SERS measurements and we calculated the coefficient of determination (R2) and linearity from the constructed calibration curve. 

The sandwich assay for *L. monocytogenes *cells in milk samples was applied after careful optimization of experimental parameters. Furthermore, comparison of results obtained from SERS method and the counting was made. Dilutions of samples were conducted in buffer (PBS) for the plate-counting method, and a 100 μL sample was plated on CHROMagar Listeria agar and incubated at 37 °C for 24 h. 

## 3. Results and discussion

### 3.1. Fabrication of the nanoparticles

In our previous work, we constructed the sandwich immunoassay concerning bacteria enumeration with DTNB-labelled rod-shaped gold nanoparticles. The rod-shaped gold nanoparticles are commonly used to conduct SERS measurements. Interaction of gold nanoparticles with target analyte resulted in increasing of sensitivity in SERS measurement [37].

### 3.2. Determination of nanoparticles’ antimicrobial activities

The determination of nanoparticles’ antimicrobial activities was given in Table 1. The liveness rates were found about 95% for *L. monocytogenes*, *S. aureus* and *S. typhimurium *strains. 

**Table 1:** Antimicrobial activities of magnetic Au-nanospheres on L. monocytogenes, S. typhimurium and S. aureus.

Bacteria strains	Live bacteria amount (cfu/mL)	Live bacteria amount afternanoparticle interaction (cfu/mL)	Liveness (%)
L. monocytogenes ATCC 7644	6.8 × 107	6.5 × 107	96
S. typhimurium BAST01	7.5 × 107	7.2 × 107	96
S. aureus ATCC 25923	10.4 × 1010	9.9 × 1010	95

### 3.3. Determination of nanoparticles’ noncompetitive capturing efficiencies

In order to determination of the noncompetitive capturing efficiencies of nanoparticles were performed as shown in Table 2. 

**Table 2:** The noncompetitive capturing efficiencies of magnetic Au-nanospheres on L. monocytogenes, S. typhimurium and S. aureus.

Noncompetitive attachment of avidin modificated magnetic Au-nanospheres			
Bacteria strains		Live bacteria amount(cfu/mL)	Capturingefficiency (%)
L. monocytogenesATCC 7644	a	2.8 × 107	20
	b	5.7 × 106	
S. typhimuriumBAST01	a	4.4 × 107	2
	b	8.0 × 105	
S. aureusATCC 25923	a	9.6 × 1010	0.7
	b	6.9 × 108	
Noncompetitive attachment on immunoassay model			
Bacteria strains		Live bacteria amount(cfu/mL)	
L. monocytogenesATCC 7644	a	2.8 × 107	75
	b	2.1 × 107	

The noncompetitive attachment of avidin bound magnetic Au-nanospheres’ capturing efficiencies were found as 20%, 2% and 0.7% for *L. monocytogenes*, *S. aureus* and *S. typhimurium* strains, respectively. Noncompetitive attachment on immunoassay model for *L. monocytogenes *was found as 75%.

### 3.4. Determination of nanoparticles’ competitive capturing efficiencies

In order to determination of the competitive capturing efficiencies, 2 experiments were performed, and the obtained results were given in Table 3. 

**Table 3:** The competitive capturing efficiencies of magnetic Au-nanospheres in the A medium. (L. monocytogenes and S. typhimurium) and B medium (L. monocytogenes and S. aureus).

Competitive attachment of avidin modificated magnetic Au-nanospheres			
Bacteria strains		Live bacteria amount(cfu/mL)	Liveness ratio ( L. monocytogenes /other bacteria)
L. monocytogenesATCC 7644S. aureusATCC 25923	a	5.8 × 107	2.1
	b	2.7 × 107	
L. monocytogenesATCC 7644S. typhimuriumBAST01	a	4.5 × 107	1.9
	b	2.4 × 107	
Competitive attachment on immunoassay model			
Bacteria strains		Live bacteria amount(cfu/mL)	Liveness ratio (L. monocytogenes /other bacteria)
L. monocytogenesATCC 7644S. aureusATCC 25923	a	6.8 × 107	2.3
	b	2.9 × 107	
L. monocytogenesATCC 7644S. typhimuriumBAST01	ab	5.4 × 1072.1 × 107	2.6

In the first study, the capturing amounts of avidin coated nanoparticles (uncoated with L. monocytogenes antibody) of mixed cultures where *L. monocytogenes* and *S. typhimurium* were present in the A medium and *L. monocytogenes* and *S. aureus* were in the B medium were investigated. The liveness ratios (*L. monocytogenes*/other bacteria) were found as 2.1 in medium A and 1.9 in medium B. 

In the other study, the capturing amounts of *L. monocytogenes* antibody modified nanoparticles of mixed cultures where *L. monocytogenes* and *S. typhimurium* were present in the A medium and *L. monocytogenes* and *S. aureus* were in the B medium were investigated. The liveness ratios were found as 2.3 in medium A and 2.6 in medium B. 

### 3.5. Detection of bacteria capturing on the magnetic Au nanoparticles with SEM and TEM measurements

**Figure 1:**
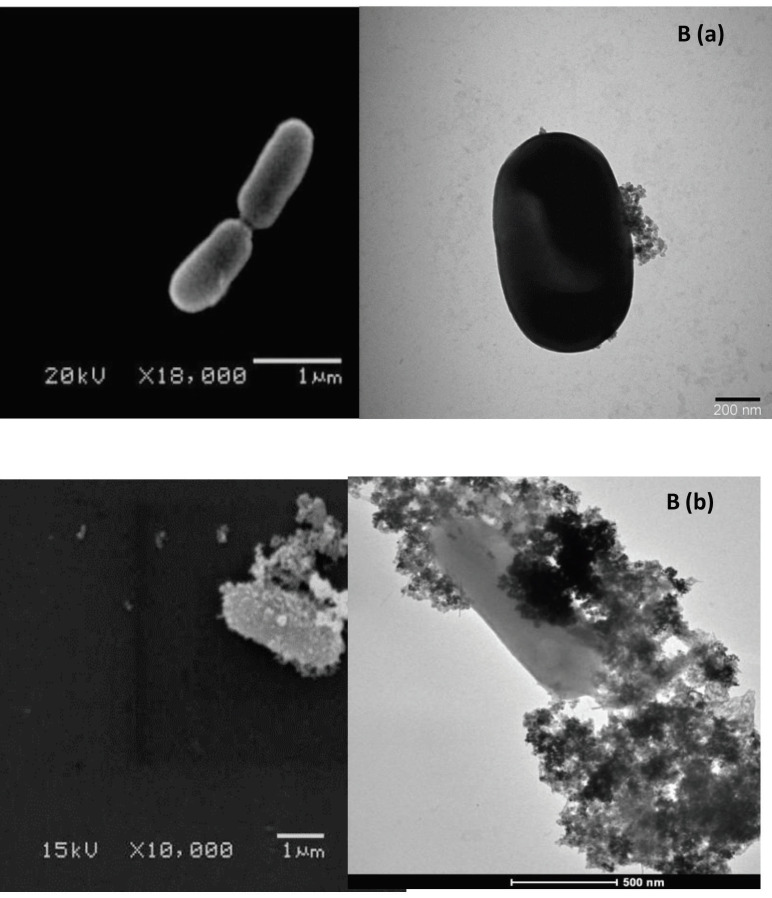
(A) Scanning electron micrograms of (a) control (L. monocytogenes without nanoparticle), (b) immunoassay model with L. monocytogenes, (B) transmission electron micrograms of (a) control (L. monocytogenes without nanoparticle) (b) immunoassay model with L. monocytogenes.

In the present study, SEM and TEM imaging of (a) control (*L. monocytogenes* without nanoparticle), (b) immunoassay model with *L. monocytogenes *were taken to verify and confirm the interactions between bacteria and nanoparticles as shown in Figures 1A and B.

### 3.6. Enumeration of L. monocytogenes using SERS

**Figure 2:**
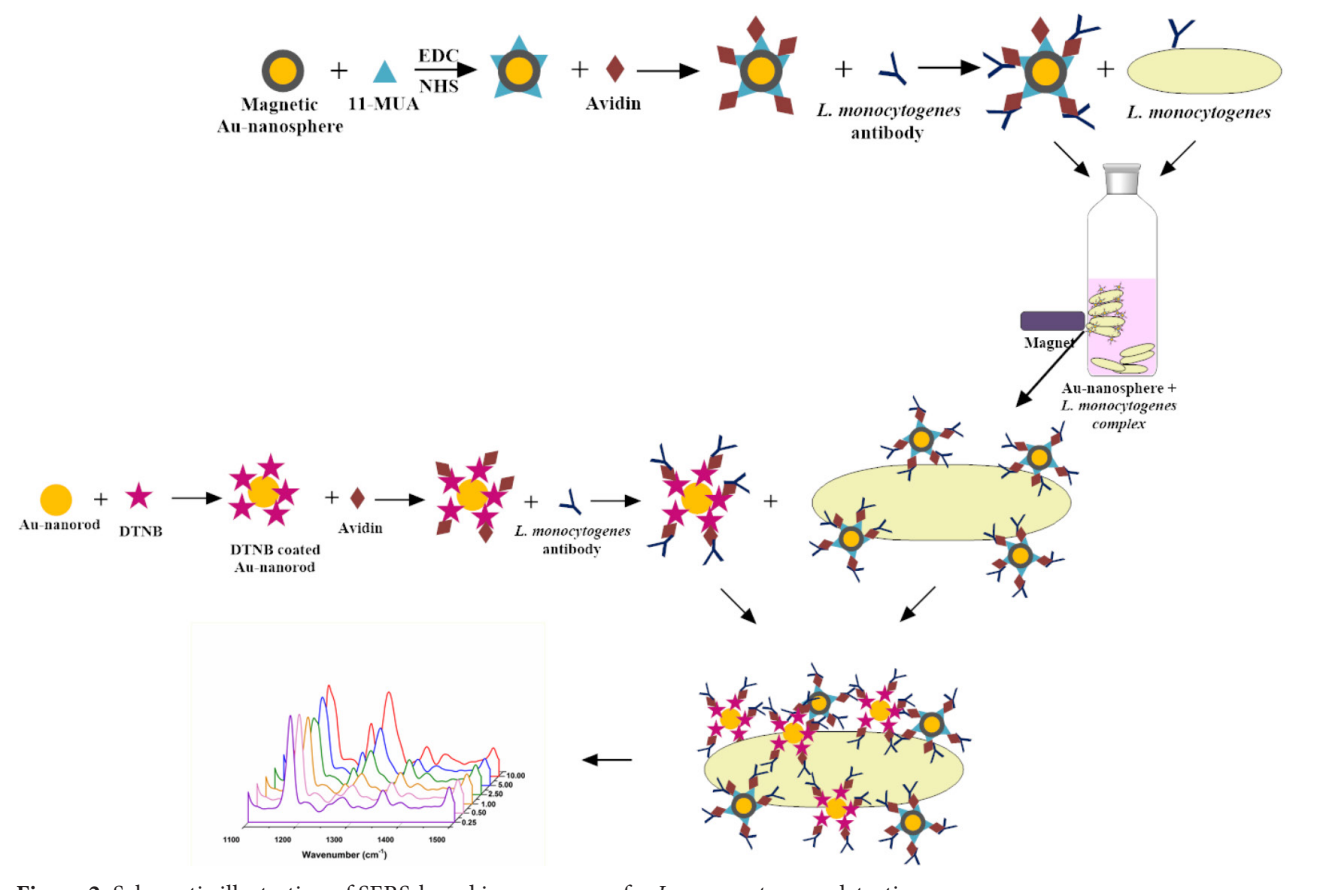
Schematic illustration of SERS-based immunoassay for L. monocytogenes detection.

Shown in Figure 2, the stepwise immunoassay strategy was proposed in this study and we focused on the selective detection of *L. monocytogenes*as SERS based diagnostic test. The proposed method was evaluated in terms of analytical performance. The presented solution is similar to the SERS assay described in our previous studies, but the main advantages of the present assay for enumeration of *L. monocytogenes*is simplicity due to the elimination of sophisticated sample preparation procedures especially in milk samples. It is also provided that there is no interference from sophisticated milk matrix on *L. monocytogenes *enumeration using SERS based assay. 

**Figure 3:**
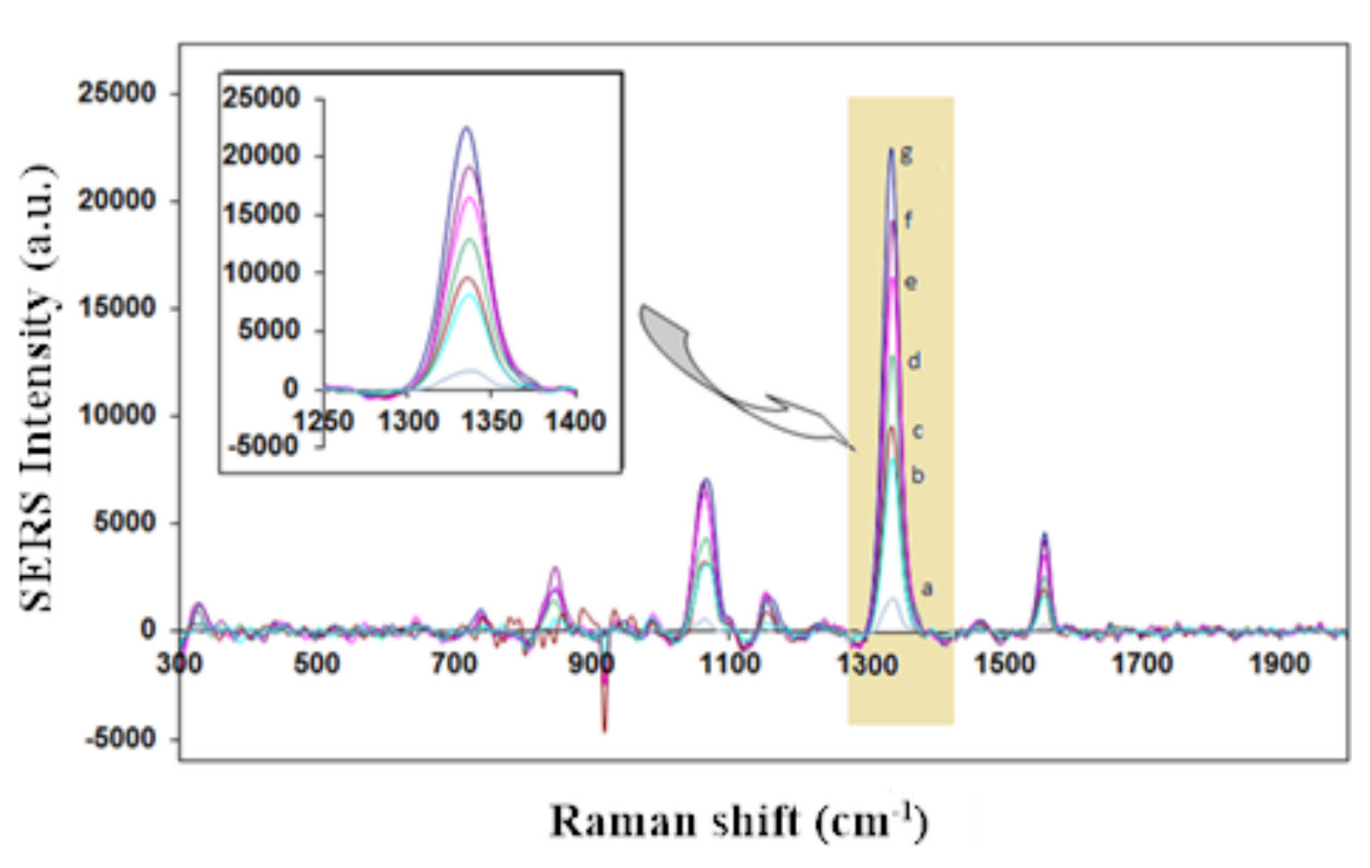
Symmetric NO2 stretching bands of DTNB range from 2.2 × 101 to 2.2 × 106 cfu/mL L. monocytogenes concentration in SERS-based sandwich immunoassay; L. monocytogenes concentrations of a) no Listeria monocytogenes, b) 2.2 × 101 cfu/mL, c) 2.2 × 102 cfu/mL, d) 2.2 × 103 cfu/mL, e) 2.2 × 104 cfu/mL, f ) 2.2 × 105 cfu/mL, g) 2.2 × 106 cfu/mL.

**Figure 4:**
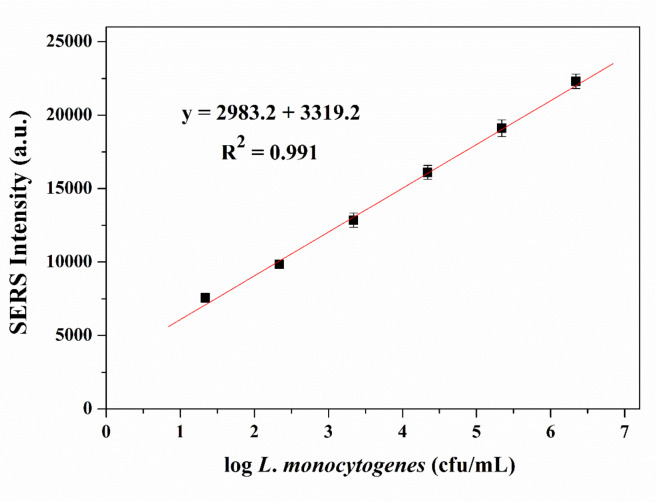
Calibration curve for target oligonucleotide sequence in a range from 2.2 × 101 to 2.2 × 106 cfu/mL in SERS-based immunoassay.

The SERS spectra for *L. monocytogenes* assays detection method conducted by using gold nanorod as a Raman tag label shows good response with the addition of various concentrations of *L. monocytogenes* as shown in Figure 3. We observed the symmetric nitro group stretching at 1336 cm-1which is attributable to the DTNB as reporter molecule.SERS peak intensity were measured to quantify *L. monocytogenes*. We performed to construct the calibration curve with the various concentrations of *L. monocytogenes* (from 2.2 × 101 to 2.2 × 106 cfu/mL). As shown in Figure 4, with the increase of different concentrations of *L. monocytogenes, *we observed the increase of SERS signal intensity. The SERS signal tracks with *L. monocytogenes *population density and becomes distinguishable in the presence of 101 bacteria. It is also mentioned that a good linear correlation (R2 = 0.991) was obtained between 102–106 cfu/mL* L. monocytogenes* concentration. We calculated the limit of detection and limit of quantification values as 12 cfu/mL and 37 cfu/mL, respectively. 

The accuracy of the proposed assay was obtained using milk samples by the SERS-immunoassay and compared with plate-counting methods as shown in Table 4. 

**Table 4:** Comparison of the results obtained for the analysis of milk samples by the SERS-immunoassay and classical counting methods.MethodsConcentrations (cfu/mL)SERS-immunoassay3.6 × 1013.9 × 1024.5 × 1036.0 × 1044.8 × 105Classical counting3.8 × 1014.0 × 1024.2 × 1035.2 × 1044.5 × 105

Methods	Concentrations (cfu/mL)				
SERS-immunoassay	3.6 × 101	3.9 × 102	4.5 × 103	6.0 × 104	4.8 × 105
Classical counting	3.8 × 101	4.0 × 102	4.2 × 103	5.2 × 104	4.5 × 105

It was found that the results procured by the presented immunoassay method and the plate counting method were very similar. 

## 4. Discussion

*L. monocytogenes* species are among the crucial foodborne pathogens that cause disease in humans and animals. This species can be found especially in milk and dairy products. In this study, the immunosensor model was developed to detect *L. monocytogenes* in mixed culture media. In literature, many rapid analysis methods were developed for *L. monocytogenes* detection. Alhogail et al. designed colorimetric biosensor to detect rapidly the amidolytic activity of Listeria protease [38]. The detection limit was found to be 2.17 × 102 cfu/mL in milk and meat samples. Another study was performed from Zhang et al. using Fe3O4 nanoparticle cluster which possesses high efficient peroxidase-like activity with a 5.2 × 103 cfu/mL detection limit [39]. The other study was based on fluorescence assay using aptamer-conjugated magnetic nanoparticles [40]. The detection limit of 102 cfu/mL of *L. monocytogenes* was obtained. To the best of our knowledge, this is the first report in which a IMS-SERS based assay was utilized to detect *L. monocytogenes.* The analytical parameters of nanoparticle coated *L. monocytogenes *sensor were investigated and the developed immunosensor was found as quite selective for *L. monocytogenes*. A linear correlation between population density of *L. monocytogenes *and SERS signal intensity was found from 2.2 × 101 to 2.2 × 106 cfu/mL and LOD was found to be 12 cfu/mL. Also, *L. monocytogenes* was detected easily in milk samples and the results generated by the SERS-immunoassay were comparable with the reference plate-counting methods. Thereby, the assay was very promising for monitoring and enumeration of bacteria in complex matrices such as milk.

## Acknowledgment

The authors gratefully acknowledge the financial support from the Gazi University Research Fund through Grant No. 46/2010-02.

## Conflict of interest

All authors disclose no conflict of interest that may have influenced either the conduct or the presentation of the research.

## Informed consent

Manuscripts reporting the results of experimental investigations did not conduct with humans.

This study was presented at the Taiwan-Turkey Science Summit entitled “Translation of Cells, Nanomaterials and Signaling Molecules into Regenerative Medicine” between April 1 to 3, 2018.
